# Integrative analyses of gene expression and DNA methylation profiles in breast cancer cell line models of tamoxifen-resistance indicate a potential role of cells with stem-like properties

**DOI:** 10.1186/bcr3588

**Published:** 2013-12-19

**Authors:** Xue Lin, Jian Li, Guangliang Yin, Qian Zhao, Daniel Elias, Anne E Lykkesfeldt, Jan Stenvang, Nils Brünner, Jun Wang, Huanming Yang, Lars Bolund, Henrik J Ditzel

**Affiliations:** 1 Sino-Danish Breast Cancer Research Centre, Copenhagen, Denmark; 2 Department of Biomedicine, University of Aarhus, Bartholins Allé 6, DK-8000 Aarhus, Denmark; 3 BGI-Shenzhen, Beishan Industrial Zone, Yantian District, Shenzhen 518083, China; 4 Department of Cancer and Inflammation Research, Institute of Molecular Medicine, University of Southern Denmark, J. B. Winsloews Vej 25.3, DK-5000 Odense, Denmark; 5 School of Life Science and Technology, Tongji University, 1239 Siping Road, Shanghai 200092, China; 6 Danish Cancer Society Research Center, Strandboulevarden 49, DK-2100 Copenhagen, Denmark; 7 Department of Veterinary Disease Biology, Faculty of Health and Medical Sciences, University of Copenhagen, Strandboulevarden 49, DK-2100 Copenhagen, Denmark; 8 James D. Watson Institute of Genome Sciences, Hangzhou, China; 9 Department of Oncology, Odense University Hospital, Søndre Boulevard 29, DK-5000 Odense, Denmark

## Abstract

**Introduction:**

Development of resistance to tamoxifen is an important clinical issue in the treatment of breast cancer. Tamoxifen resistance may be the result of acquisition of epigenetic regulation within breast cancer cells, such as DNA methylation, resulting in changed mRNA expression of genes pivotal for estrogen-dependent growth. Alternatively, tamoxifen resistance may be due to selection of pre-existing resistant cells, or a combination of the two mechanisms.

**Methods:**

To evaluate the contribution of these possible tamoxifen resistance mechanisms, we applied modified DNA methylation-specific digital karyotyping (MMSDK) and digital gene expression (DGE) in combination with massive parallel sequencing to analyze a well-established tamoxifen-resistant cell line model (TAM^R^), consisting of 4 resistant and one parental cell line. Another tamoxifen-resistant cell line model system (LCC1/LCC2) was used to validate the DNA methylation and gene expression results.

**Results:**

Significant differences were observed in global gene expression and DNA methylation profiles between the parental tamoxifen-sensitive cell line and the 4 tamoxifen-resistant TAM^R^ sublines. The 4 TAM^R^ cell lines exhibited higher methylation levels as well as an inverse relationship between gene expression and DNA methylation in the promoter regions. A panel of genes, including *NRIP1*, *HECA* and *FIS1,* exhibited lower gene expression in resistant vs. parental cells and concurrent increased promoter CGI methylation in resistant vs. parental cell lines. A major part of the methylation, gene expression, and pathway alterations observed in the TAM^R^ model were also present in the LCC1/LCC2 cell line model. More importantly, high expression of *SOX2* and alterations of other *SOX* and *E2F* gene family members, as well as RB-related pocket protein genes in TAM^R^ highlighted stem cell-associated pathways as being central in the resistant cells and imply that cancer-initiating cells/cancer stem-like cells may be involved in tamoxifen resistance in this model.

**Conclusion:**

Our data highlight the likelihood that resistant cells emerge from cancer-initiating cells/cancer stem-like cells and imply that these cells may gain further advantage in growth via epigenetic mechanisms. Illuminating the expression and DNA methylation features of putative cancer-initiating cells/cancer stem cells may suggest novel strategies to overcome tamoxifen resistance.

## Introduction

Around 80% of breast cancer patients present with primary breast tumors that are estrogen receptor (ER) alpha-positive, suggesting that the tumor is dependent on estrogen for growth [1,2]. Accordingly, most of these patients are offered endocrine therapy, which currently consists of the anti-estrogen tamoxifen or aromatase inhibitors. These drugs can be used successfully both in the adjuvant and advanced disease settings. Tamoxifen belongs to the selective ER modulator class of drugs that act both as antagonists and as agonists in an ER-dependent and tissue-dependent manner [3]. For example, in breast cancer tissue, tamoxifen acts as a competitive estrogen antagonist by competing with estrogen for binding to ER, thereby inhibiting the growth of estrogen-dependent breast cancer cells [4]. However, about one-third of primary ER-positive breast tumors do not benefit from adjuvant tamoxifen treatment, resulting in disease recurrence [5]. In metastatic disease, disease progression eventually occurs in most patients receiving tamoxifen treatment.

Acquired endocrine resistance is suggested to develop as a result of a complex set of molecular changes, including specific gene expression alterations, and/or modifications and loss of ER [6]. These changes have been observed in *in vitro* models of tamoxifen resistance and in ER-positive breast cancer patients with recurrent disease following endocrine treatment [7]. As it is currently not possible to predict sensitivity/resistance to endocrine treatment in ER-positive breast cancer patients, new tests to identify endocrine-resistant ER-positive breast cancer are being developed using different molecular markers [8].

Several distinct molecular mechanisms may lead to tamoxifen resistance, and within individual tumors different cancer cells may use different mechanisms, complicating the evaluation of tamoxifen resistance mechanism(s) when examining whole tumor samples. These obstacles have led to studies of isogenic tamoxifen-resistant breast cancer cell line model systems that may have some advantages in pinpointing individual resistance mechanisms. The estrogen-responsive and tamoxifen-sensitive human breast cancer cell line MCF-7 [9,10] and its derived tamoxifen-resistant sub-lines MCF-7/TAM^R^-1, MCF-7/TAM^R^-4, MCF-7/TAM^R^-7 and MCF-7/TAM^R^-8 [11,12] constitute a well-established *in vitro* model that has been used to identify several proteins potentially involved in signaling pathways associated with tamoxifen resistance in ER-positive breast cancer cells; for example, phosphorylated Akt, PKCα, PKCδ, EGFR and HER2 [13-15]. A number of these proteins have been positively validated in clinical studies [16-18]. Tamoxifen resistance in the TAM^R^ cell lines was developed by culturing the parental cell line in an initial high dose of tamoxifen (1 μM). In contrast, tamoxifen resistance in the LCC1/LCC2 cell line model system was developed by incrementally increased doses of tamoxifen to the parental cell line MCF7/LCC1 (estrogen independent and tamoxifen sensitive) [19].

Epigenetic alterations, which include modifications of DNA, histones and chromatin, play an important role in transcription regulation. Epigenetic changes are reversible and can occur quickly during environmental changes [20]. Increasing evidence indicates that these epigenetic alterations, particularly DNA methylation, may be used as future markers for diagnosis, prognosis and prediction of response to therapies [21]. A few studies have suggested that epigenetic alterations may also play a role in tamoxifen resistance in breast cancer [22,23]. Recently, cancer stem cells were also reported to be associated with cancer therapy resistance [24]. There are thus three hypotheses in the development of tamoxifen resistance: first, ER-positive breast cancer cells can acquire tamoxifen resistance by epigenetic alternation resulting in changed mRNA expression of genes pivotal for estrogen-dependent growth; second, tamoxifen resistance develops due to selection of preexisting cancer initiating cells/cancer stem-like cells; and third, tamoxifen resistance results from a combination of the above hypotheses – that is, by selection of preexisting resistant cells that gain or repress gene expression to acquire further advantage in growth via epigenetic mechanisms, such as changed DNA methylation.

To test the above hypotheses, we applied modified DNA methylation-specific digital karyotyping (MMSDK) [25] and digital gene expression (DGE) in combination with next-generation parallel sequencing to analyze methylation and gene expression profiles of the parent MCF-7 breast cancer cell line and its tamoxifen-resistant TAM^R^ cell lines (see Additional file 1 for a description and an illustration of the MMSDK methods). The resulting methylation data were compared with the corresponding gene expression profiles. In addition, methylation and gene expression alterations identified in the TAM^R^ cell line model were validated in the LCC1/LCC2 tamoxifen-resistant cell line model.

## Methods

### TAM^R^ cell line model

The MCF-7 human breast cancer cell line was originally received from The Breast Cancer Task Force Cell Culture Bank, Mason Research Institute (Worcester, MA, USA). The MCF-7 cells were gradually adapted to grow in low serum concentration (initially 0.5% fetal calf serum (FCS) and 1% FCS after phenol red was omitted from the culture medium [11]), and the tamoxifen-sensitive sub-line MCF-7/S0.5 [26] was used to establish tamoxifen-resistant (TAM^R^) cell lines by extended treatment with 1 μM tamoxifen, as described previously [11,26]. The four TAM^R^ cell lines, MCF-7/TAM^R^-1 (TAM^R^-1), MCF-7/TAM^R^-4 (TAM^R^-4), MCF-7/TAM^R^-7 (TAM^R^-7) and MCF-7/TAM^R^-8 (TAM^R^-8), were derived from distinct colonies that emerged in cultures of MCF-7/S0.5 cells treated with 1 μM tamoxifen [11,27]. The TAM^R^ cell lines were maintained in Dulbecco’s modified Eagle’s medium/F12 (1:1) supplemented with 1% FCS and 1 μM tamoxifen, as detailed by Thrane and colleagues [28]. Tamoxifen had a weak agonistic effect (20 to 80% increase after 5 days) on growth of the tamoxifen-resistant cell lines [28]. Withdrawal of tamoxifen for up to 15 weeks did not change the growth characteristics of the TAM^R^-1 cell line, demonstrating a stable resistant phenotype [11]. The cells were kept within 10 passages throughout the experiment to reduce possible variability between experimental results.

### LCC1/LCC2 cell line model

The estrogen-independent, but tamoxifen-responsive, LCC1 cell line was established from the hormone-dependent parent cell line MCF-7 through prolonged withdrawal from potent estrogenic stimuli both *in vivo* and *in vitro*[29]. The *in vivo* selected cell line was further passaged in ovariectomized athymic nude mice and re-established *in vitro* to generate a new cell line, MCF-7/LCC1, which is also estrogen independent but is similarly tamoxifen responsive as its parent cell line [30]. Furthermore, the new cell estrogen-independent, tamoxifen-resistant sub-line LCC2 [19] was developed through growth of LCC1 in incrementally increased dosages of tamoxifen *in vitro*. LCC1 and LCC2 were cultured in Dulbecco’s modified Eagle’s medium/F12 without phenol red, supplemented with dextran charcoal-stripped 5% FCS and 1% penicillin/streptomycin. The cells were maintained at 37°C in a humidified atmosphere of 95% ambient air and 5% carbon dioxide. Genomic DNA and total RNA were isolated from LCC1 and LCC2.

### Modified DNA methylation-specific digital karyotyping

For optimized MMSDK library construction, the *Bss*HII restriction enzyme (New England Biolabs, Hitchin, UK) was selected. This enzyme has 52,167 recognition sites in the human genome, but only unmethylated sites are cleaved. The sites are preferentially located in CpG islands and promoters in the human genome, thus providing higher resolution for mapping DNA methylation in the human genome than previous methods [25].

*In silico* digital digestion of the unmethylated human genome with *Bss*HII and *Nla*III was performed. The distribution of the lengths of the theoretically generated *Bss*HII/*Nla*III *f*ragments was calculated and the majority of fragments were shorter than 1,000 base pairs (bp), with a frequency peak at 50 to 150 bp. Within CpG islands (CGIs), 23,818 *Bss*HII recognition sites were identified, accounting for 45.7% of all *Bss*HII recognition sites in the human genome. Our approach also allowed determination of the methylation state of CpGs in repeat sequences. According to RepeatMasker [31], 23.0% of the *Bss*HII sites were located within repeat sequences in the human genome. MMSDK libraries using *Bss*HII/*Nla*III were generated from the parental tamoxifen-sensitive cell line MCF-7/S0.5 and the four TAM^R^ cell lines TAM^R^-1, TAM^R^-4, TAM^R^-7 and TAM^R^-8. DNA was isolated from the cell lines using a DNeasy® Blood & Tissue Kit (Qiagen, Manchester, UK) according to the manufacturer’s protocol. Genomic DNA was digested with *Bss*HII followed by ligation to biotinylated adaptors and fragmented by *Nla*III (New England BioLabs) cleavage. Because *Bss*HII only cuts unmethylated regions, binding of DNA fragments to streptavidin-conjugated magnetic beads allows separation of unmethylated and methylated fragments. Bound DNA was ligated to another adaptor N containing the *Mme*I restriction enzyme recognition site, and then digested with *Mme*I (New England Biolabs), which generates short sequence tags (16 to 17 bp, due to enzyme cut floating).

The resulting tags were ligated with another adaptor P7 and amplified by polymerase chain reaction (PCR) with primers N and P7 for 18 cycles. The five indexed MMSDK libraries were sequenced in one lane, resulting in 1.38 Gb clean tag data for all five cell lines, with an average sequencing amount of ~270 Mb per library. A description of the MMSDK method is provided in Additional file 1. Prior to normalization, the total number of aligned tags of MMSDK for MCF-7/0.5, TAM^R^-1, TAM^R^-4, TAM^R^-7 and TAM^R^-8 were 1,908,177, 2,574,465, 2,556,778, 2,884,094 and 2,650,408, respectively. On average, 59.5% of the tags with mapping quality ≥20 were mapped back to the simulated *Bss*HII/*Nla*III reference library, which was used for the subsequent analysis.

### Digital gene expression tag sequencing

DGE libraries were generated from MCF-7/S0.5, the four TAM^R^ cell lines, and the LCC1 and LCC2 cell lines. Total RNA was extracted from the cell lines with TRI Reagent (Sigma, Brondby, Denmark) according to the manufacturer’s protocol. The integrity of the extracted RNA was verified by agarose gel electrophoresis, and the concentration of RNA was estimated by spectrophotometry. Subsequently, mRNA was separated from total RNA by poly-T-coated beads and converted to cDNA. The cDNA was subjected to *Nla*III digestion followed by N-ligation, *Mme*I digestion, P7 ligation and PCR to prepare a DGE library in a manner analogous to that in MMSDK. The PCR products containing tags from MMSDK and DGE have the same size and structure since they were generated with the same enzymes and procedures. Additionally, an index (barcode) system was developed to allow multiplexed sequencing of samples for tag profiling through incorporation of barcode sequences into the sequences of the adaptor P7.

The PCR products were purified and pooled for direct sequencing with Hiseq 2000 (Illumina, San Diego, CA, USA) using standard single-end 50-nucleotide sequencing. The sequences of the adaptors and primers are available in Additional file 2. The five indexed DGE libraries were sequenced in one lane, resulting in 1.71 Gb clean tag data for all five TAM^R^ cell lines, with an average sequencing amount of ~340 Mb per library. Similarly, the two indexed DGE libraries for LCC1 and LCC2 were sequenced in another lane. Prior to normalization, the total number of aligned tags of DGE for MCF-7/0.5, TAM^R^-1, TAM^R^-4, TAM^R^-7, TAM^R^-8, LCC1 and LCC2 were 2,164,460, 2,038,646, 2,047,000, 2,111,546, 1,980,773, 1,583,224 and 3,096,827, respectively. On average, 40.8% of the tags with mapping quality ≥20 were mapped back to the simulated *Nla*III human transcriptome (refMrna reference library), which were used for the subsequent analysis.

### Accession numbers

The raw data and metadata of DGE and MMSDK for the MCF-7/S0.5 and four TAM^R^ cell line model were deposited in the NCBI Gene Expression Omnibus database [GEO:GSE40665].

### Statistical and bioinformatic analysis for MMSDK and DGE

#### Identifying and trimming reads (tags)

According to the experimental design, tags of 16 to 17 nucleotides were mapped together with the neighboring four nucleotides (the recognition sequence of *Nla*III) to *in silico* references to reveal the methylation status using MMSDK analysis, and to reveal the mRNA profile using DGE analysis. The command line tool FASTX-Toolkit implemented in Perl was used to trim the adaptor sequence [32]. The trimmed tags were subjected to quality filtering so that only tags with sequencing quality >30 for >80% of the nucleotides were used for subsequent analysis.

#### Mapping tags

For tag mapping, we generated a simulated *Bss*HII reference library by *in silico* enzyme digestion of the human genome regardless of the methylation state*.* This library was used as a reference for subsequent mapping of the tags in the MMSDK analysis. In the DGE analysis, refMrna (hg19; University of California, Santa Cruz (UCSC), CA, USA) was subjected to *in silico* digestion with *Nla*III and *Mme*I and the digested mRNAs were used as a reference for mapping cDNA tags. Subsequently, the Burrows-Wheeler Alignment tool (BWA) procedure [33] allowing one mismatch for aligning the MMSDK and DGE tags to the simulated *Bss*HII reference library and the refMrna reference library, respectively, was applied.

For the MMSDK analysis, the genomic locations used to assess methylation levels were annotated based on the genomic information of the simulated *Bss*HII reference library, and the methylation status of each *Bss*HII site was used to represent the corresponding genomic region in which this *Bss*HII site was located. The count of the tags representing a particular *Bss*HII site is a measure of its degree of nonmethylation in the genome; that is, the smaller the tag count, the higher the level of methylation of the site in question. For the DGE analysis, the count of the tags represents the gene expression level; that is, the higher the tag count, the higher the expression level. After mapping and annotating the tags, the data were normalized by equalizing the total number of tags for all samples in MMSDK and DGE, respectively. The normalized data were used for the subsequent analysis.

#### Visualization of MMSDK and DGE data

Integrative Genomics Viewer was used to visualize the differences between individual tamoxifen-resistant cell lines and the parental tamoxifen-sensitive cell line MCF-7/S0.5 with regard to the MMSDK and DGE data [34]. Normalized MMSDK (total 51,918 genomic loci) and DGE tag (total 19,070 genes) features were used for visualization. The hg19 human genome was used as a reference [35]. We defined gene promoters as the regions located in the upstream 2 kb from transcript starting sites (TSSs) and the first exon. We adopted the same criteria (GC content >50%, ratio of the observed CpGs to the expected CpGs >0.6, length >200 bp) used by the UCSC Genome Browser for the definition of CGIs.

### Principle component analysis and unsupervised cluster analysis

Qlucore Omics Explorer 2.3 software (Qlucore, Sweden, Lund) was used to perform principle component analysis. Normalized MMSDK (total 51,918 genomic loci) and DGE tag (total 19,070 genes) data were used as input data for principle component analyses without filtering. An unsupervised hierarchical clustering analysis was applied to analyze the similarities in MMSDK and DGE profiles across the five TAM^R^ cell model lines using Qlucore Omics Explorer 2.3 software with a data filter requiring that the variance/maximum variance of variables across samples is higher than 0.001. A total of 17,561 genomic loci and 5,220 transcripts passed the filter for unsupervised cluster analyses, respectively. The Pearson correlation algorithm was employed for similarity metric calculation. Average linkage clustering was chosen to organize samples in a tree structure.

### Pathway and enrichment analysis

Ingenuity Pathways Analysis software (Ingenuity Systems, Redwood City, CA, USA) was used to perform pathway analysis and uncover related networks for these genes. Genes showing >2-fold alterations in expression between the MCF-7/S0.5 and TAM^R^ cell lines were selected as input data in the first analysis. In the second analysis, only genes exhibiting altered gene expression and inverse altered methylation levels were included. In addition, an enrichment analysis using gene set enrichment analysis [36] to identify over-represented pathways and genes was performed on genes exhibiting >2-fold alterations in expression between MCF-7/S0.5 and TAM^R^ cell lines.

### Reduced representation of bisulfite sequencing

Gemomic DNA (5 μg) from LCC1 and LCC2 was digested by the *Msp*I restriction enzyme, (500 U/per sample; New England BioLabs) overnight at 37°C, and a Mini Purification kit (Qiagen) was used to purify the digested products. End repair was performed, adding A and adaptors, where the cytosines in the paired end adaptor sequence were methylated. The ligated product was subjected to size selection in 2% agarose gel (Bio-RAD, Richmond, CA, USA) at 100 V for 2 hours. Agarose gel bands with 150 to 240 bp (according to insert DNA size 40 to 120 bp) and 240 to 340 bp (according to the ligated target DNA size 120 to 220 bp), for example, were excised and two libraries were generated from each sample (one consisting of 40 to 110 bp target sequences and the other of 110 to 220 bp target sequences). DNA from the two excised gel pieces was recovered by Gel Extraction Purification Kit (Qiagen), followed by bisulfite treatment using a EZ DNA Methylation-Gold kit (Zymo Research, Freiburg, Germany). The resulting converted DNA was amplified by PCR and, following purification, the reduced representation of bisulfite sequencing (RRBS) libraries were generated by performing paired-end 50-nucleotide sequencing with Hiseq 2000 (Illumina, San Diego, CA, USA). The adaptor sequences were filtered out from the subsequent analysis and the resulting reads were aligned using Bismark software [37]. Only uniquely mapped reads with restriction enzyme cutting sites at the 5′ end were used for subsequent methylation analyses. The sequencing depth and percentage of methylated cytosines/total investigated cytosines for each C location were calculated. The genomic annotation information was based on the hg19 human genome [35]. Gene promoters and CGI were defined using the same criteria as for the MMSDK analysis. According to the genomic annotation and coordinates, DNA methylation information between the TAM^R^ cell line model (MMSDK data) and the LCC1/LCC2 cell line model (RRBS data) were compared.

### Quantitative reverse transcriptase-PCR validation of gene expression

Quantitative reverse transcriptase-PCR was performed using the 2^–ΔΔCt^ method [38]. Briefly, total RNA was extracted and subjected to DNase I (RNase-free) digestion (Life Biotechnologies, Paisley, UK) to exclude contamination from genomic DNA. Subsequently, 1 μg purified total RNA was reverse-transcribed in a final volume of 20 μl containing 10 μl 2× reverse transcriptase buffer (dNTPs and MgCl_2_), 1 μl random hexamers (300 ng/μl) and 2 μl M-MuLV RNase H + reverse transcriptase (DyNAmo Capillary SYBR Green two-step quantitative reverse transcriptase-PCR kit; Finnzymes, Thermo Fisher Scientific, Slangerup , Denmark). cDNA synthesis was conducted by incubation at 25°C for 10 minutes (primer extension), 37°C for 30 minutes, 85°C for 5 minutes (reaction termination) and 4°C hold (sample cooling). Either *β*_*2*_*-microglobulin* or *pumilio homolog 1* (*PUM1*) was used as the internal control for normalization of the data [38]. The *SOX2* PCR primer sequence was obtained from Li and colleagues [39], while primers for PRKCA and PUM1 were purchased from Qiagen. The primer design for β_2_-microglobulin was performed using Primer3 [40]. Both pairs of primer sequences were blasted against UCSC Genes in UCSC Genome Bioinformatics using the In-silico PCR tool to confirm the expected unique amplification of *SOX2* and *β*_*2*_*-microglobulin* genes, respectively. The PCR primer sequences are available in Additional file 2.

The quantitative PCR reaction was composed of 2× master mix, forward and reverse PCR primer and 0.5 μl 10-fold diluted cDNA. The analysis was performed in triplicate using the LightCycler 480 system (Roche, Mannheim, Germany). A melting curve analysis was performed after PCR to confirm a single peak (unique amplification) for the PCR products, which were then run on a 2% electrophoresis agarose gel to further confirm the presence of a single band of the expected size. Accurate quantification was confirmed by generation of calibration curves by serial dilutions (native, 10-fold, 100-fold and 1,000-fold dilution) of one TAM^R^ sample and MCF-7/S0.5, which showed the same amplification efficiency of *SOX2* and *β*_*2*_-*microglobulin*, respectively. The threshold cycle (Ct) number at which the fluorescent signal is associated with an exponential increase of PCR products (by default) was used to calculate the normalized target. For each sample, Δ values were determined by subtracting the average of triplicate Ct values of the target gene (*SOX2*) from that of the reference gene (*β*_*2*_-*microglobulin* or *PUM*1). The relative gene expression level of *SOX2* and *PRKCA* in each TAM^R^ sample was normalized relative to the parental MCF-7/S0.5 cell line. The relative expression levels of the genes were determined by subtracting the average of triplicate Ct values of the target genes (*SOX2* and *PRKCA*) from that of the reference genes (*β*_*2*_-*microglobulin* or *PUM1*, respectively). Finally, the relative expression level (fold-change) of each TAM^R^ sample compared with their parental cell line (MCF-7/S0.5) was determined using 2^–ΔΔCt^, in which:

$$\begin{array}{ll} \mathrm{\Delta \Delta Ct} & ={\left(Ct_{\mathrm{gene}}-Ct_{\mathrm{reference}\;\mathrm{gene}}\right)}_{\mathrm{TAMR}}\\ & \;–{\left(Ct_{\mathrm{gene}}-Ct_{\mathrm{reference}\;\mathrm{gene}}\right)}_{\mathrm{MCF}-7/S0.5} \end{array}$$

## Results

### Visualization and integrative analysis

**Figure 1:**
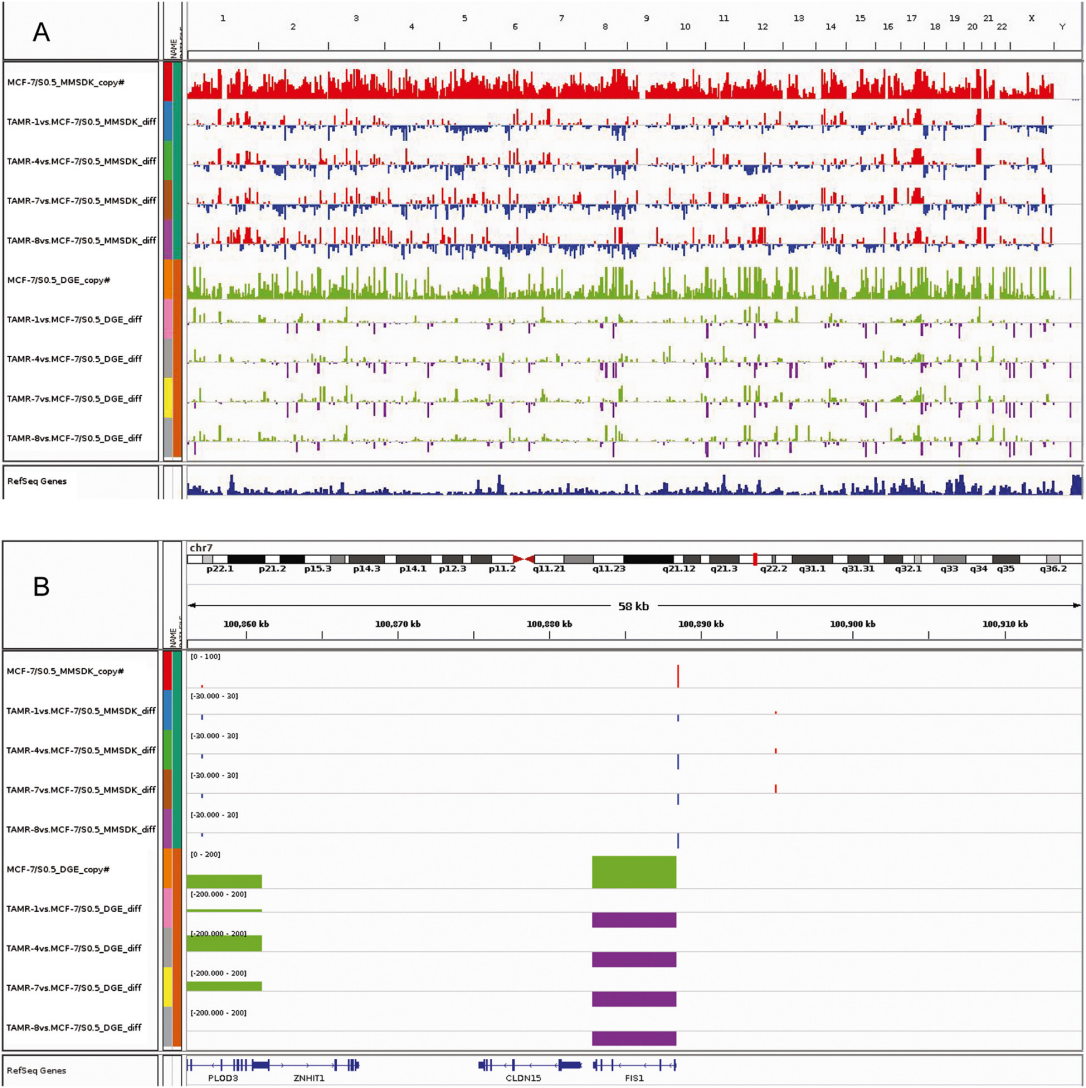
**Global landscape of the differences in modified DNA methylation-specific digital karyotyping and digital gene expression profiles for the parental MCF-7/S0.5 and the four TAM**^**R **^**cell lines as visualized by Integrative Genomics Viewer.** The *x* axis shows the locations in the whole human genome **(A)** and the region of *FIS1* on chromosome 7 **(B)**. The height of the bars in modified DNA methylation-specific digital karyotyping (MMSDK) for MCF-7/S0.5 (red) shows the extent of the number of tags representing the frequency of nonmethylated CpG islands at the particular locus. The MMSDK data for the four TAM^R^ cell lines is expressed as the difference in expression between a given TAM^R^ and the parental cell line (red/blue). The height of the bars in digital gene expression (DGE) for MCF-7/S0.5 (green) is proportional to the gene expression level. The DGE data for the four TAM^R^ cell lines are expressed as the difference in expression between a given TAM^R^ cell line and the parental cell line (green/blue).

The MMSDK and DGE profiles of the parental cell line MCF-7/S0.5 and the four TAM^R^ cell lines were initially compared and visualized by Integrative Genomics Viewer (Figure 1A), allowing a global view in a whole human genome scale of the DNA methylation (MMSDK) and gene expression (DGE) values in MCF-7/S0.5 as well as alterations between cell lines. For example, detailed information on the differences in MMSDK and DGE in the region centered with *FIS1* gene on chromosome 7 is shown in Figure 1B.

### Principal component analysis and unsupervised cluster analysis

**Figure 2:**
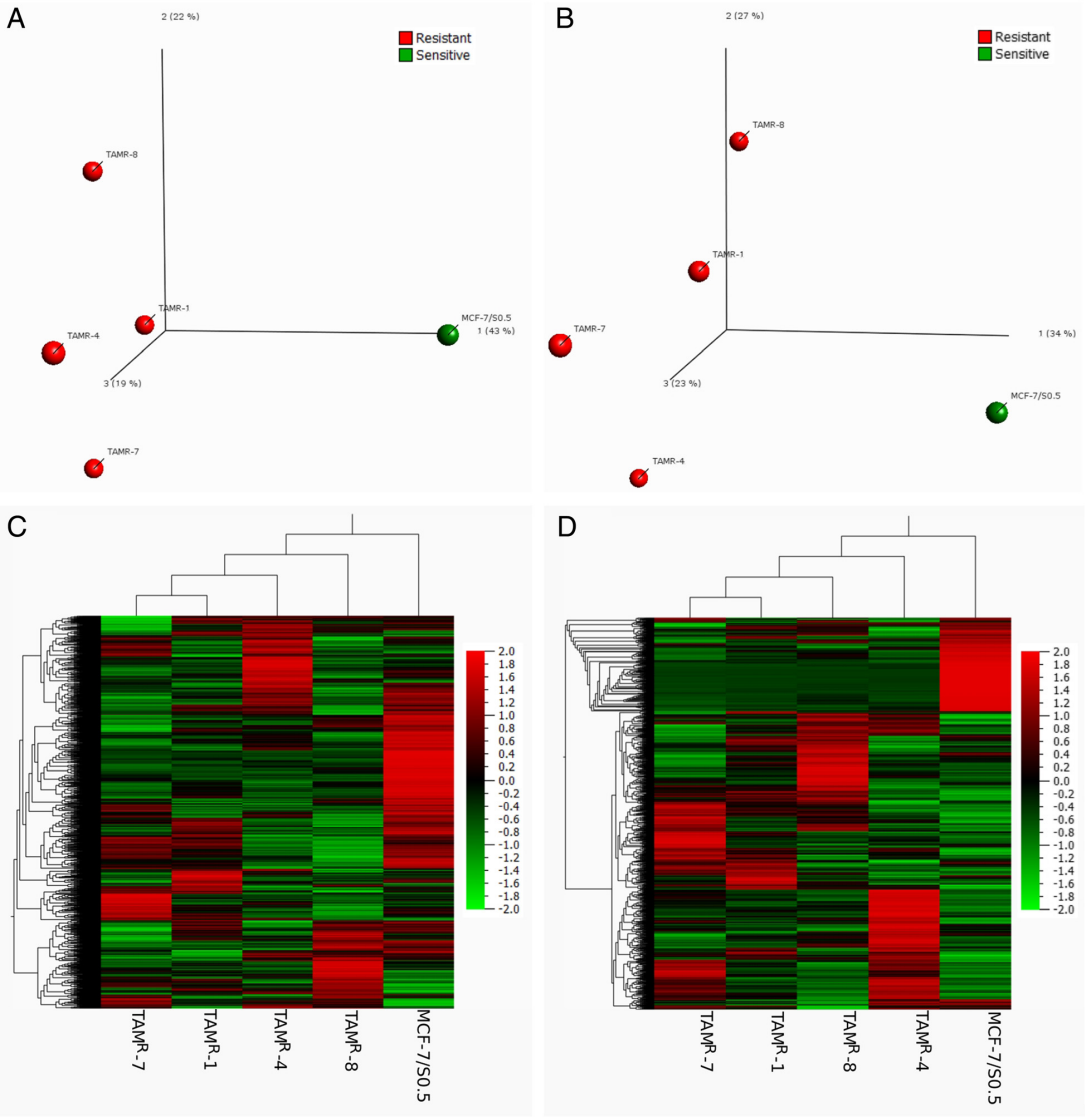
**Principle component analysis and unsupervised cluster analysis for DNA methylation and gene expression data in TAM**^**R **^**and MCF-7/S0.5 cell lines.** Principle component analysis results for modified DNA methylation-specific digital karyotyping (MMSDK) **(A)** and digital gene expression (DGE) **(B)** data show that the TAM^R^ cell lines grouped separately from the parental MCF-7/S0.5 cell line. The unsupervised cluster analyses of MMSDK **(C)** and DGE **(D)** also show clear separation of TAM^R^ cell lines from the MCF-7/S0.5 cell line.

Principle component analysis of the MMSDK data, which depicts all variables without any *a priori* classification and data filtering in the three-dimensional space, showed that MCF-7/S0.5 separated from the four TAM^R^ cell lines, indicating overall differences in global DNA methylation profiles between parental and resistant cell lines (Figure 2A). The four TAM^R^ cell lines also separated from each other, but to a lesser extent than from the parental cell line. Similarly, principle component analysis of the DGE data demonstrated a clear separation of MCF-7/S0.5 from the four TAM^R^ cell lines (Figure 2B). In unsupervised cluster analysis, MCF-7/S0.5 also separated from the four TAM^R^ cell lines for both MMSDK (Figure 2C) and DGE (Figure 2D).

### Overview of DNA methylation alterations between parental and tamoxifen-resistant sub-lines

**Figure 3:**
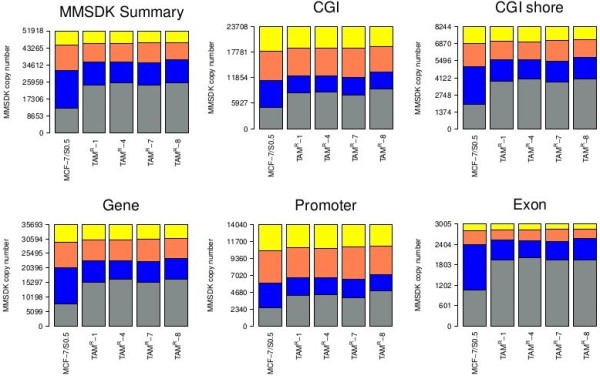
**Distribution of DNA methylation levels of different genomic components in MCF-7/S0.5 versus TAM**^**R **^**cell lines.** MCF-7/S0.5 shows low DNA methylation levels compared with TAM^R^ cell lines in both the global profile and the different genomic components (CpG island (CGI), CGI shore, gene, promoter and exon). The *x* axis shows the color-coded methylation states of CpGs for the MCF-7/S0.5, TAM^R^-1, TAM^R^-4, TAM^R^-7 and TAM^R^-8 cell lines. The mean methylation state of CpGs is categorized into very high (grey, 0 to 1 tag), high (blue, 2 to 10 tags), intermediate (orange, 11 to 100 tags), and low (yellow >100 tags). The *y* axis shows the proportion of CpGs covered by methylation scores at low, intermediate, or high levels. Coordinates for genomic features were taken from the University of California, Santa Cruz genome database, with the exception of CGI shores, which were defined as 2 kb on either side of the CGI.

DNA methylation analysis revealed that the four TAM^R^ cell lines exhibited globally higher DNA methylation levels than MCF-7/S0.5. The distribution of the genomic loci in different genomic components (counting the number of tags from given components) is shown in Figure 3 and Additional file 3. The annotation of the genomic components is from UCSC. Notably, across all genomic components as well as in the global view, the four TAM^R^ cell lines showed higher DNA methylation levels compared with their parental tamoxifen-sensitive cell line.

### Genes exhibiting altered expression between parental and tamoxifen-resistant sub-lines in the TAM^R^ cell line model

Initially, we investigated the expression levels of *ESR1*, *ESR2*, *PGR*, *IGF1R*, *PTEN*, *ERBB2* (HER2), *PRKCA* and *NOTCH3*, which were previously implicated in tamoxifen resistance. There was no significant difference in the expression of *ESR1*, but slightly increased expression of *ESR2* (2.3-fold) was observed. *ERRB2* (3.8-fold), *PRKCA* (2.6-fold) and *NOTCH3* (6.9-fold) also exhibited increased expression in TAM^R^ versus MCF-7/S0.5 cell lines, while expression of *PGR* (−32.1-fold), *IGF1R* (−3.7-fold) and *PTEN* (−10.2-fold) was decreased. Generally, these results using DGE tag sequencing were consistent with those of previous studies [11,41,42]. The slight difference in the expression levels of *ESR1* and *ESR2* in the current study compared with previous studies could be due to differences in methodologies. Further investigation of key cell cycle genes such as *MYC* and *CCND1* (cyclin D_1_) showed that these genes remained highly expressed in all resistant sub-lines, but there was no significant difference (*MYC* 1.0-fold) and only slightly lower levels (*CCND1* –1.8-fold change) in TAM^R^ versus MCF-7/S0.5 cell lines.

Next, we investigated the expressed genes that exhibited >2-fold altered expression common for all TAM^R^ cell lines versus MCF-7/S0.5 and identified 3,063 genes, of which 1,561 were expressed at higher levels (Additional file 4) and 1,502 at lower levels (Additional file 5) in TAM^R^ cell lines versus MCF-7/S0.5.

**Figure 4:**
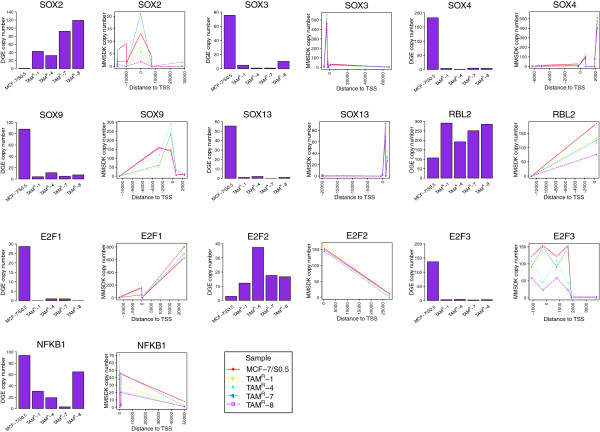
**Relationship between the state of DNA methylation and gene expression for individual genes.** Gene expression and DNA methylation states around transcription start sites (TSSs) are presented for selected genes. To the left of each panel, the height of the bar represents the gene expression level (normalized tag number of digital gene expression (DGE) data), while the line in the right panels shows DNA methylation levels for the genomic loci around TSSs.

Interestingly, several of the altered genes related to pluripotency and differentiation, including *SOX2,* which exhibited higher expression levels (74.8-fold) in TAM^R^ cell lines versus MCF-7/S0.5 (Figure 4). The whole *SOX* gene family was further studied and showed decreased expression of *SOX3* (−17.3-fold), *SOX4* (−51.6-fold), *SOX9* (−12.8-fold) and *SOX13* (−54.3-fold) in TAM^R^ cell lines versus MCF-7/S0.5 (Figure 4), while the remaining *SOX* genes were not expressed or exhibited very low expression in both TAM^R^ cell lines and MCF-7/S0.5 (data not shown). We also observed alterations of the expression of *E2F* gene family, with decreased expression of *E2F1* (−57.6-fold) and *E2F3* (−44.9-fold) and elevated expression of *E2F2* (7.3-fold) in TAM^R^ cell lines versus MCF-7/S0.5, while expression of *E2F4* was not significantly altered (1.6-fold). The expression levels of *E2F1*, *E2F2* and *E2F3* were considerably lower than *E2F4* in all cell lines. Since *E2F* interacts with RB-related pocket proteins (p130 and p105), we also investigated the expression of these two pocket protein genes. *RBL2* (p130) exhibited higher expression than *NFKB1* (p105) in all cell lines. Further, *RBL2* and *FOXA1* also showed higher expression (2.4-fold and 2.9-fold, respectively), while *NFKB1* showed decreased expression (−3.2-fold) in TAM^R^ cell lines versus MCF-7/S0.5. Taken together, altered expression of pluripotency and differentiation genes, including increased expression of *SOX2*, decreased expression of other *SOX* gene families, and alterations of the expression of *E2F* genes and pocket protein genes, may suggest a role for cancer-initiating cells/cancer stem-like cells in tamoxifen resistance.

### Relationship between DNA methylation and gene expression in parental and tamoxifen-resistant sub-lines in the TAM^R^ cell line model

Initially, we delineated the global impact of DNA methylation on gene expression by classifying all genes into three groups based on gene expression levels: low (0 to 1 tag); intermediate (2 to 50 tags); and high (>50 tags). Accordingly, DNA methylation loci were also classified into four groups according to methylation levels: very high (0 to 1 tag); high (2 to 10 tags); intermediate (11 to 70 tags); and low (>71 tags).

**Figure 5:**
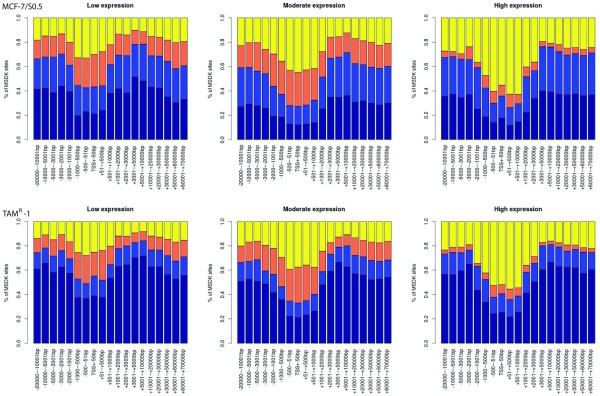
**Relationship between DNA methylation and gene expression in MCF-7/S0.5 and TAM**^**R**^**1.** An inverse relationship between DNA methylation and gene expression levels is noted. The expressed genes are grouped according to expression levels: low (left), moderate (middle) and high (right). Dark blue, light blue, orange and yellow present extreme-high, high, medium and low DNA methylation levels. The *x* axis shows the genomic location relative to the transcriptional start site. The *y* axis shows the percentage of methylation for a given genomic location.

Since the impact of DNA methylation on gene expression is known to depend on the genomic location relative to the TSS, plots were generated showing the global positional relationship between DNA methylation and gene expression at different expression levels (Figure 5 and Additional file 6). The plots demonstrate a relationship between DNA methylation and distance to TSS locations, with the lowest DNA methylation level being at the TSS region across all gene expression levels. Comparing DNA methylation levels between the groups showed an inverse relationship between gene expression and DNA methylation levels; that is, higher methylation levels were associated with lower gene expression levels. Second, we investigated in detail the relationship between DNA methylation and gene expression in individual genes of interest. Figure 4 shows plots of mRNA expression, DNA methylation and genomic location for genes of interest, including the *SOX* gene family (Figure 4).

A panel of 44 genes were found to exhibit higher promoter CGI DNA methylation (twofold change) in TAM^R^ cell lines versus MCF-7/S0.5 cells, with concurrent lower gene expression (twofold change) in TAM^R^ cell lines versus MCF-7/S0.5. Among these genes, *NRIP1*, *HECA* and *FIS1* were of particular interest since they have previously been reported to be associated with breast cancer pathogenesis. Another set of 18 genes exhibited lower promoter CGI DNA methylation (twofold change) in TAM^R^ cell lines versus MCF-7/S0.5 cells, with concurrent higher gene expression (twofold change) in TAM^R^ cell lines versus MCF-7/S0.5. The DNA methylation state of promoters with and without CGIs for the genes that exhibited altered expression in TAM^R^ cell lines versus MCF7/S0.5 is listed in Additional files 7 and 8.

### Pathway analysis

To further elucidate the pathways affected in connection with tamoxifen resistance in breast cancer, we performed pathway analysis of genes exhibiting altered methylation of promoter sites in TAM^R^ versus MCF-7/S0.5 cells and concurrent inverse alteration of gene expression levels using the Ingenuity Pathway Analysis software (Ingenuity Systems, Redwood City, CA, USA). Among the genes with significant DNA methylation loci, we observed significant enrichment of genes associated with cell cycle, cellular growth and proliferation, including *FOS*, *LMNA*, *RUNX1*, *SLC9A3R1*, *SNTB2*, *STAT5B*, *SUZ12*, *UGCG*, *VEGFA*, *AK4*, *NCOA6*, *NCOR2*, *SOX4*, *EPB41L1*, *EHD1* and *SNTB2*. This suggests an important role in tamoxifen resistance of epigenetic alteration of genes involved in growth and proliferation of cancer cells. Similarly, analysis of differentially expressed genes identified by mRNA sequencing showed significant enrichment of genes associated with cell cycle, cellular assembly and organization, DNA replication, cell survival and death as well as cell proliferation. These genes included *BACE1*, *CADM1*, *CCNA2*, *CDC42SE1*, *CDKN2C*, *CDKN3*, *CDT1*, *CENPE*, *CKS2*, *COL7A1*, *CTGF*, *DAAM1*, *ERBB2*, *ERRFI1*, *GLO1*, *LAMP2*, *MKI67*, *MLXIP*, *MYBL1*, *MYBL2*, *MYO10*, *NEK2*, *OSMR*, *POLE2*, *PRC1*, *RAB31*, *RAD51AP1*, *RALB*, *RHOD*, *SOLH*, *SOX4*, *TGFB1*, *THBS1*, *WNT5B* and *ZWINT*. The canonical pathways with significant gene enrichment included the RAR activation and the DNA damage response pathways. In addition, pathways such as Notch, Wnt/β-catenin and transforming growth factor beta signaling, which are known for extensive cross-talk and are implicated in stemness, were shown to be associated with genes that showed differential expression patterns between TAM^R^ and MCF-7/S0.5 cells. Finally, gene set enrichment analysis of our expression data demonstrated enrichment of the pluripotency and differentiation processes, as well as the E2F family (Additional file 9). *E2F1*, *E2F2*, *F2F3*, *RBL2* (p130) and *NFKB1* (p105) were enriched in the gene sets of KEGG_PANCREATIC CANCER and KEGG_CELL_CYCLE using the gene set enrichment analysis database. *SOX3* and *SOX4* were enriched in the gene set MASSARWEH_TAMOXIFEN_RESISTANCE_DN.

### Validation of methylation, gene expression and pathway alterations in the LCC1/LCC2 cell line model

**Figure 6:**
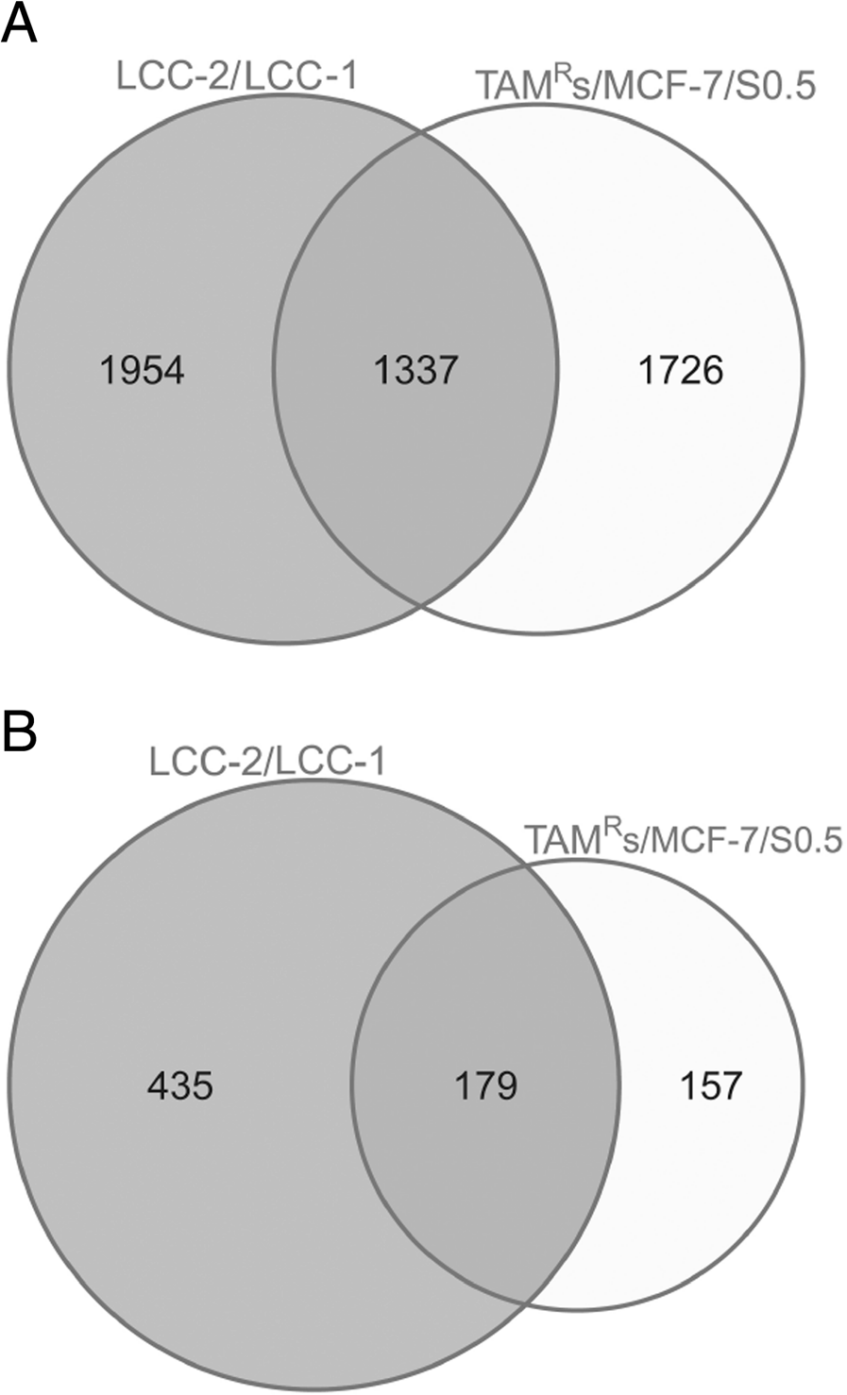
**Overlap between methylation and gene expression alterations in the TAM**^**R **^**and LCC cell line models. (A)** Venn diagram showing the overlapping number of genes that exhibited altered expression (twofold change) in both the TAM^R^ versus MCF-7/S0.5 and the LCC2 versus LCC1 cell line models. **(B)** Venn diagram showing the overlapping number of genes that exhibit altered promoter CpG island (CGI) DNA methylation (twofold change) and concurrent inversely altered gene expression (twofold change) in both the TAM^R^ versus MCF-7/S0.5 and the LCC2 versus LCC1 cell line models.

To ensure that the alterations observed in TAM^R^ cell lines versus MCF-7/S0.5 cells were not unique to this specific cell line model, we examined whether the methylation, gene expression and pathway alterations associated with TAM^R^ could be observed in another tamoxifen-resistant cell line model, LCC1/LCC2. DNA methylation analysis of LCC1 and LCC2 was performed by RRBS, and the results were compared with the DNA methylation profile of TAM^R^ according to genomic coordinates. The two cell line models, in general, exhibited global inherited DNA methylation profiles, reflecting their biological origins. In addition, the two cell line models shared several DNA methylation alterations. Further, many genes that exhibited altered gene expression in the TAM^R^ cell line model inversely correlated with DNA methylation and were also identified in the LCC1/LCC2 cell line model (Figure 6 and Additional files 10 and 11). Some important genes, such as *PGR*, *CCND1*, *MYC*, *PTEN*, *SOX4*, *SOX13* and *TGFβ1*, and pathways such as transforming growth factor beta signaling that are implicated in tamoxifen resistance in the TAM^R^ cell line model were also identified in the LCC1/LCC2 cell line model.

### Validation of SOX2 and PRKCA gene expression

**Figure 7:**
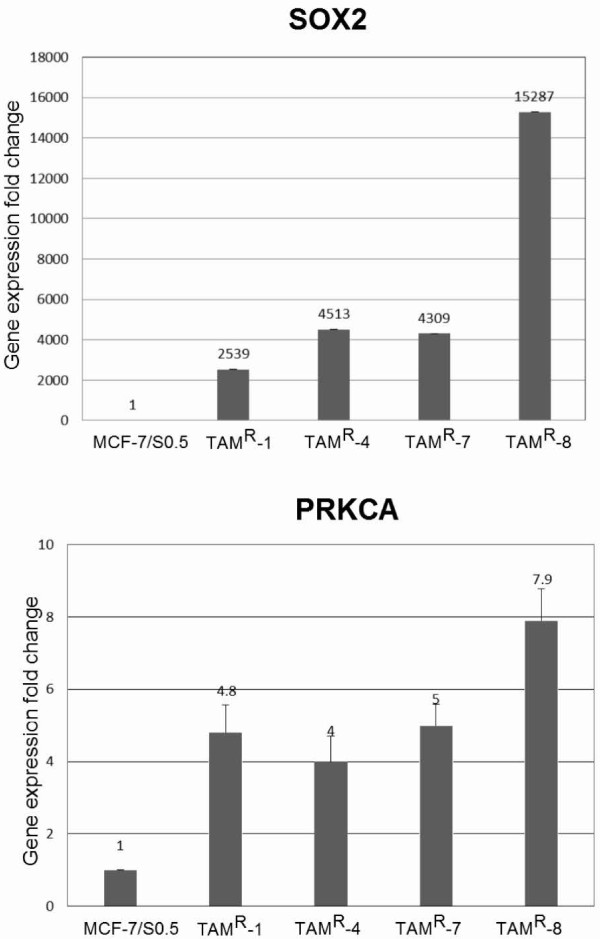
**Relative expression of *SOX2 *and *PRKCA *in the MCF-7/S0.5 and TAM**^**R **^**cell lines.** Comparison of the relative expression of *SOX2* and *PRKCA* genes (fold-changes) in the four TAM^R^ cell lines compared with their parental cell line MCF7/S0.5.

The higher gene expression levels of *SOX2* and *PRKCA* in TAM^R^ cell lines versus MCF-7/S0.5 obtained by sequencing were further evaluated by quantitative reverse transcriptase-PCR and confirmed that the relative expression of both *SOX2* and *PRKCA* in all four TAM^R^ cell lines was significantly higher than in MCF-7/S0.5 (Figure 7). The expression level of *SOX2* was particularly high in TAM^R^-8.

## Discussion

Tamoxifen has a great impact on clinical management of breast cancer; however, about one-third of early-stage breast cancer patients eventually experience disease recurrence and subsequent mortality [7]. Resistance to tamoxifen is thus a major clinical issue and considerable efforts have been made to elucidate the mechanisms leading to this resistance, including decrease or loss of ERα expression that could result from mutations of the *ESR1* gene, and/or hypermethylation of the *ESR1* gene promoter, altered expression of ERβ protein, endocrine adaptation, pharmacologic tolerance (for example, increased metabolism of tamoxifen to agonistic metabolites), altered patterns of co-regulator (co-activator and co-repressor) expression, cross-talk between ERα and growth factor signaling pathway, or influence of the phosphoinositide 3-kinase cell survival pathway and interaction between ER protein with the stress-activated protein kinase/c-junNH2 terminal kinase pathway [6,7].

Several distinct mechanisms may lead to tamoxifen resistance, and within individual tumors different cancer cells may use different mechanisms, complicating evaluation of tamoxifen resistance mechanisms in whole tumor samples. To simplify the matter, we used a cell line model wherein ER-positive MCF-7/S0.5 cells were exposed to high-dose tamoxifen resulting in tamoxifen-resistant TAM^R^ cell lines. Our TAM^R^ cell lines seem to mimic the clinical situation wherein tumors are exposed to high doses of tamoxifen that eradicate the majority of cells, but a few cells may survival and lead to relapse and therapy failure. In addition, an independent tamoxifen-resistant cell line model (LCC1/LCC2), which mimics another clinical situation wherein tumors are exposed initially to insufficient dosages of tamoxifen, was used to verify the finding in the TAM^R^ cell line model. Our next-generation sequencing of mRNA from both cell line models revealed that many genes associated with cancer stem cells exhibit altered expression in resistant versus parental cell lines. Increasing evidence supports the hypothesis that these resistant cells arise from putative cancer-initiating cells or cancer stem-like cells. For example, tamoxifen treatment in combination with targeted cancer stem cell inhibition achieves a better outcome than tamoxifen treatment alone, indicating that surviving cancer stem-like cells may remain viable after initial endocrine therapy [24]. *In situ* observations have identified candidate cells with stem cell-like features of various phenotypes in breast cancer samples [24], and it has been suggested that such cells may be responsible for therapeutic failures [43-45].

In our study, we found high expression of *SOX2* in the TAM^R^ cell lines. *SOX2* is a transcription factor essential for maintaining self-renewal of undifferentiated normal embryonic stem cells, and also plays an important role in cancer development and recurrence [46]. In addition, *SOX2* is one of the four factors that, by induction, can induce pluripotent stem cells from mouse embryonic or adult fibroblasts [47]. In fact, the expression of the *SOX2* gene in itself could be responsible for stem cell properties [46]. *SOX2* has been shown to be expressed in early-stage breast tumors, while expression of other normal stem cell markers, such as *OCT4* or *NANOG*, was not observed. Furthermore, the expression of *SOX2*, but not *OCT4* or *NANOG*, induced mammosphere formation in cultures, underscoring the possibility that increased expression of *SOX2* is sufficient to induce cancer stem cell properties [46]. Interestingly, a recent study showed that TAM^R^ cells exhibited increased mammosphere-forming capability compared with MCF-7/S0.5 cells (8% vs. 3%) [48]. Additionally, the promoting role of *SOX2* in cell proliferation mediated through *CCND1* (cyclin D_1_) has been demonstrated by gain-of-function and loss-of-function experiments using MCF-7 cells [48]. The positive correlation of the co-expression of *SOX2* and *CCND1* with tumorigenesis has also been demonstrated in clinical breast cancer samples [49].

In contrast to *SOX2*, several other members of the *SOX* family (*SOX3*, *SOX4*, *SOX9* and *SOX13*) showed decreased expression in the resistant versus parental cell lines. These *SOX* gene family members play important roles in differentiation and tissue maturation [50], and have also been implicated in regulating β-catenin activity [51-54]. Since the majority of *SOX* genes negatively regulate Wnt/β-catenin signaling, their expression (in contrast to *SOX2*[55]) could suppress the activity of cyclin D_1_. Decreased expression of these genes could thus attenuate their suppressing effect on proliferation. Taken together, *SOX2* and the other *SOX* family members activate the expression of *MYC* and *CCND1*, perhaps bypassing the blocked ER-mediated mitogenesis by which cancer cell proliferation can be maintained.

We also identified alterations in the expression of the *E2F* gene family, which strengthens the association of stemness features with the development of tamoxifen resistance. The *E2F* gene family of transcription factors provides important downstream effector functions in a pathway that controls the expression of genes involved in cell cycle progression, G_1_/S transition and DNA replication [56]. Becker and colleagues demonstrated that human stem cells differ from somatic cells in the expression of members of the *E2F* family and RB-related pocket proteins [57]. They reported that human stem cells and teratocarcinoma cells show a selective reduction in the expression of *E2F1*, *E2F2*, *E2F3* and p105 (encoded by *NFKB1*) and enhanced expression of *E2F4*, *E2F5*, *E2F6* and p130 (encoded by *RBL2*) compared with human normal somatic IMR90 cells [57]. In our study, decreased expression of *E2F1 E2F3* and *NFKB1* (p105) and increased expression of *RBL2* (p130) was observed in the tamoxifen-resistant versus parental cell lines. Moreover, *RBL2* (p130) showed higher expression levels than *NFKB1* (p105), and *E2F4* showed higher expression levels than *E2F1*, *E2F2* and *E2F3* in all five cell lines. This observation further supports the role of cancer-initiating cells/cancer stem-like cells in the development of resistance to tamoxifen treatment.

*NOTCH3*, which has been shown to play a role in maintenance of stemness in breast cancer cells, was also more highly expressed in the tamoxifen-resistant versus parental cell lines. *NOTCH3* has been shown to be upregulated when normal breast tissue is grown as mammospheres [58], and downregulation of *NOTCH3* by short hairpin RNA interference in MCF-7 cells reduced the capacity of first-generation mammospheres to produce a second generation [59]. *NOTCH3* was also found to be upregulated in CD44^+^ populations of normal cells and breast cancer cells [60].

In addition to gene expression alterations, we also determined DNA methylation levels in the resistant and parental cell lines using MMSDK. In a global view, our data show that high DNA methylation in the neighborhood of transcription start sites correlated with lower gene expression. A large panel of genes was found to exhibit higher promoter CGI DNA methylation in the resistant versus parental cells and concurrent lower gene expression in the resistant versus parental cells. Among these genes, *NRIP1*, *HECA* and *FIS1* were of particular interest because they have previously been reported to be associated with breast cancer pathogenesis [61-63], and further studies of these genes will be pursued. Our results differ somewhat from those of an earlier study that examined the gene expression and methylation status of a single tamoxifen cell line [22]. For example, Fan and colleagues found that their tamoxifen-resistant cell line was associated predominantly with global promoter hypomethylation relative to the parental line [22], while, in contrast, we observed global hypermethylation of all four tamoxifen-resistant versus parental cell lines. However, one should note that the tamoxifen-resistant cell line generated by Fan and colleagues [22] was derived from a different strategy than our four tamoxifen-resistant cell lines, and the technology used to analyze gene and methylation levels also differed (array vs. sequencing).

DGE, as used in our study to investigate gene expression, is a common method that exhibits high fidelity. DGE captures the sequence from the 3′ end of transcripts, thereby avoiding involvement of complex statistical model to address isoform splicing events for estimating gene expression. MMSDK, as we used to examine the DNA methylation profiles, is also a reliable method as shown in an earlier study where the results identified by MMSDK could be validated by quantitative PCR-based and bisulfite clone sequencing [25]. In addition, to avoid putative influence of PCR amplification bias, PCR amplification was limited to a maximum of 18 cycles.

In our study, not all genes exhibiting altered gene expression also exhibited corresponding promoter methylation changes, perhaps due to the resolution of the MMSDK method that did not identify all methylation alterations. For some individual genes, the MMSDK sampling locations (*Bss*HII recognition sites) are still limited. Many *SOX* family genes and *E2F* family genes have no *Bss*HII site in their promoter and enhancer regions, limiting our analysis of methylation alterations in these genes. In addition, for many genes more than one methylation site was examined, some of which exhibited altered expression while others did not. It is not currently known which of the sites are of functional importance. Finally, some genes of interest in our study, such as *SOX2*, did not show any impact of DNA methylation on gene expression (according to *Bss*HII recognition sites on its promoter region), which does not exclude the possibility of the impact of DNA methylation status of other *cis*-regulatory element(s) on the expression of *SOX2*. Further studies are needed to confirm this hypothesis. Notably, *FOXA1*, a pioneer factor in development and differentiation [64,65], has been suggested to interact with hormonal receptors (ER and androgen receptor) and play a role in breast cancer and prostate cancer, and even in tamoxifen resistance [66-69]. Our results suggest an association between reprogramming transcription, epigenetic plasticity and tamoxifen resistance. The precise mechanism and profound role of this gene require further investigation.

## Conclusion

**Figure 8:**
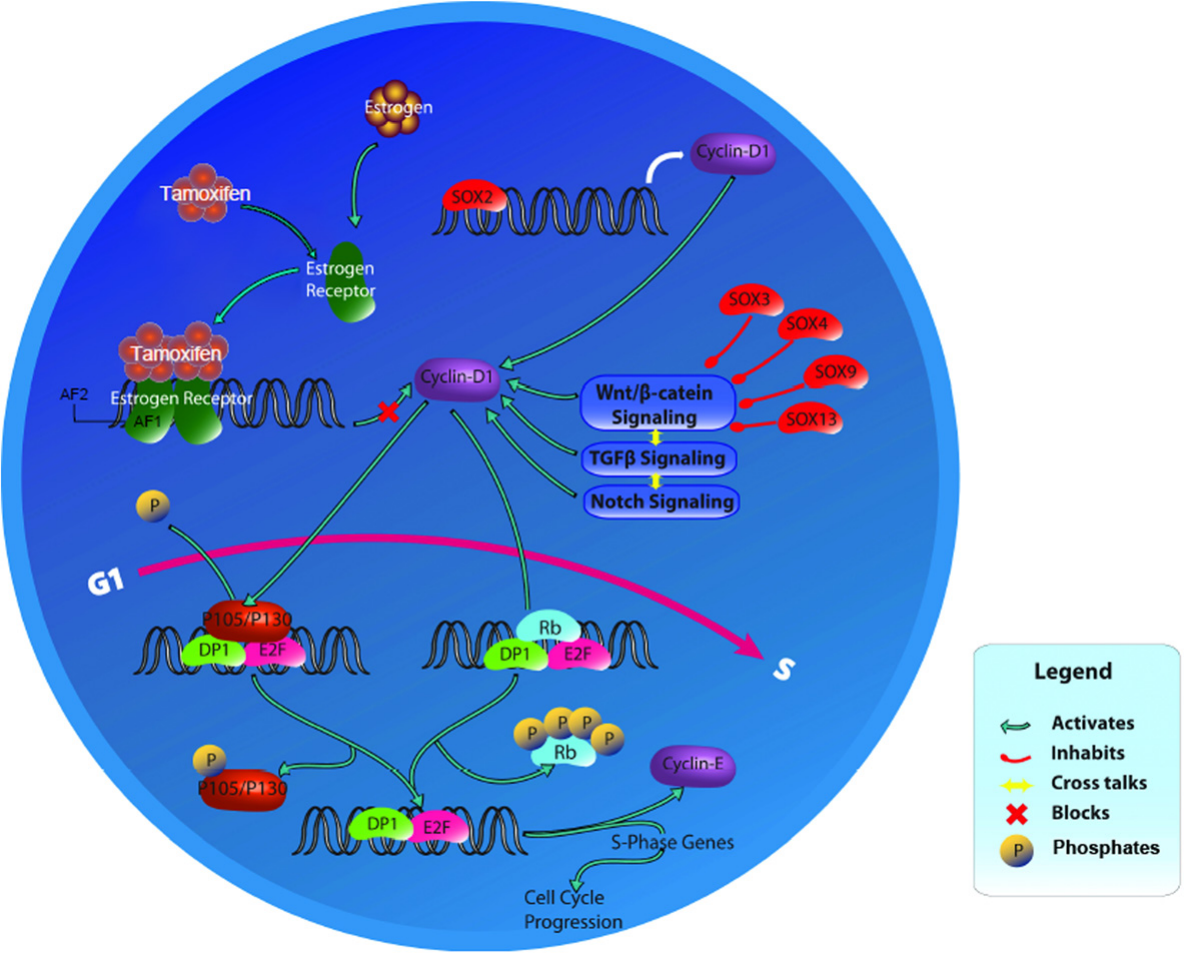
**A possible mechanism implicated in tamoxifen resistance in the TAM**^**R **^**cell line model.** While tamoxifen competitively binds with estrogen receptor (ER) and prevents binding between estradiol and ER, thereby blocking estrogen mitogenetic activity, *CCND1* (cyclin D_1_) expression remains high due to direct activation mediated by *SOX2* and/or by the Wnt/β-catenin pathway through attenuation of the suppression effect of other *SOX* gene family members on this pathway. Transforming growth factor beta (TGFβ) and Notch pathways are also implicated in activation of *CCND1* (cyclin D_1_). Cyclin D_1_ interacts with pocket proteins (Rb, P105 and P130) and abrogates their suppressive effect on *E2F*. Finally, activated E2F accomplishes G_1_/S transition. By this mechanism, the cancer cells may bypass the blocked estrogen-mediated mitogenesis and maintain proliferation.

High expression of *SOX2* and suppression of other *SOX* gene family members in combination with usage of the *E2F* gene family, RB-related pocket protein genes and highlighted stem-like cell-associated pathways implies that cancer-initiating cells/stem-like cells may be crucial for development of resistance to tamoxifen (Figure 8). Large differences in global gene expression and DNA methylation profiles between the parental MCF-7 tamoxifen-sensitive human breast cancer cell line and its high-dosage tamoxifen-selected resistant subpopulations were observed. In general, DNA methylation in promoter regions is shown to be associated with repression of gene expression, which also holds true for some genes previously associated with breast cancer development. Thus, although tumor-initiating cells/stem-like cells may be of primary importance, these cells might acquire survival advantage in gene expression via epigenetic mechanisms. However, it is difficult to prove this hypothesis because even the stemness-associated genes can be regulated by epigenetic changes and the present techniques do not allow the DNA methylation status of tamoxifen-selected resistant cells at the single cell level to determined (each sub-line TAM^R^ was developed from such single surviving cells). In this study, biological replicates were not sequenced. Although the results of DNA methylation and gene expression from the four individual tamoxifen-resistant TAM^R^ cell lines were highly consistent, further analysis using approaches with higher coverage, such as RRBS and RNA Seq, may confirm our findings.

Our results underscore the likelihood of stem cell-like resistant cells in tamoxifen resistance. The present study shows some evidence of stemness and cell plasticity in tamoxifen-resistant cells and poses a new hypothesis linking cell fate plasticity, epigenetic programming, and possible induced pluripotency processes with tamoxifen resistance. To prove our hypothesis and deepen understanding of the mechanism of drug resistance more information from genomic, epigenomic and transcriptomic analyses will be required, as well as deciphering cross-talk between these mechanisms in cancer cells.

## Abbreviations

bp: Base pair; CGI: CpG island; Ct: Threshold cycle; DGE: Digital gene expression; ER: Estrogen receptor; FCS: Fetal calf serum; MMSDK: Modified DNA methylation-specific digital karyotyping; PCR: Polymerase chain reaction; RRBS: Reduced representation of bisulfite sequencing; TAMR: Tamoxifen-resistant cell line model; TSS: Transcript starting site; UCSC: University of California, Santa Cruz.

## Competing interests

The authors declare that they have no competing interests.

## Authors’ contributions

XL and JL were involved in the planning and design of the study, performed the experimental work, sequencing data analysis and statistical analysis, and drafted the manuscript. GY and QZ assisted in the MMSDK analysis and contributed to the manuscript. DE was involved in DNA and RNA isolation, quantitative PCR analysis and critically revised the manuscript. AEL provided the tamoxifen-resistant TAM^R^ cell line model, interpreted data for the work and critically revised the manuscript. NB was involved in the planning and design of the study, provided the tamoxifen-resistant LCC1/LCC2 cell line model and contributed to the manuscript. JS, HY and JW interpreted data and critically revised the manuscript. LB and HJD were involved in the planning and design of the study, supervised the study and contributed to the writing and revision of the manuscript. All authors approved the manuscript for publication and have agreed to be accountable for all aspects of the work.

## Supplementary Material

Additional file 1 Presents a description and illustration of the MMSDK method. Click here for file

Additional file 2 Lists the sequence of adaptors and primers used in this study. Click here for file

Additional file 3 **Is a figure showing the distribution of DNA methylation levels of various genomic components in MCF-7/S0.5 versus TAMR cell lines.** MCF-7/S0.5 shows low DNA methylation levels compared with TAMR cell lines in the different genomic components (intron, LTR (long terminal repeat), SINE (short interspersed elements), LINE (long interspersed elements), LINE1, LINE2, and satellite). The *x* axis shows the color-coded methylation states of CpGs for the MCF-7/S0.5, TAMR-1, TAMR-4, TAMR-7 and TAMR-8 cell lines. The mean methylation state of CpGs is categorized into very high (gray, 0 to 1 tag), high (blue, 2 to 10 tags), intermediate (orange, 11 to 100 tags), and low (yellow >100 tags). *y* axis shows the proportion of CpGs covered by methylation scores at low, intermediate, or high levels. Coordinates for genomic features were taken from the UCSC genome database and LINEs are defined by RepeatMasker. Click here for file

Additional file 4 **Is a table listing the genes exhibiting higher expression (>2-fold) in TAM**^**R **^**cell lines versus MCF-7/S0.5.** To avoid the influence of large variance in low-expressed genes, expression levels <10 tags have been binned to 10 tags. Click here for file

Additional file 5 **Is a table listing the genes exhibiting lower expression (≤2-fold change) in TAM**^**R **^**cell lines versus MCF-7/S0.5.** To avoid the influence of large variance in low-expressed genes, expression levels <10 tags have been binned to 10 tags. Click here for file

Additional file 6 **Is a figure showing the relationship between DNA methylation and gene expression in TAM**^**R**^**-4, TAM**^**R**^**-7 and TAM**^**R**^**-8.** An inverse relationship between DNA methylation and gene expression levels is noted. The expressed genes are grouped according to expression levels: low (left), moderate (middle) and high (right). Dark blue, light blue, orange and yellow represent extreme-high, high, medium and low DNA methylation levels, respectively. The *x* axis shows the genomic location relative to the TSS. The *y* axis shows the percentage of methylation for a given genomic location. Click here for file

Additional file 7 **Is a table listing the genes exhibiting lower expression (≤2-fold change) and concurrent higher DNA methylation (≤2-fold change in MMSDK data) in the promoter CGI region in TAM**^**R **^**cell lines versus MCF-7/S0.5.** Click here for file

Additional file 8 **Is a table listing the genes exhibiting higher expression (>2-fold change) and concurrent lower DNA methylation (>2-fold change in MMSDK data) in the promoter CGI region in TAM**^**R **^**cell lines versus MCF-7/S0.5.** Click here for file

Additional file 9 Is a table listing the gene set enrichment analysis identifying over-represented pathways. Click here for file

Additional file 10 **Is a table listing the genes exhibiting altered expression (upregulated or down regulated, absolute value >2-fold change) in both the TAM**^**R **^**versus MCF-7/S0.5 and the LCC2 versus LCC1 cell line models.** Click here for file

Additional file 11 **Is a table listing the genes exhibiting altered expression (upregulated and down regulated, absolute value >2-fold change) and concurrent inversely altered DNA methylation in the promoter region in both the TAM**^**R **^**versus MCF-7/S0.5 and the LCC2 versus LCC1 cell line models.** Click here for file
