# Supplementary material for: Integrative analyses of gene expression and DNA methylation profiles in breast cancer cell line models of tamoxifen-resistance indicate a potential role of cells with stem-like properties
Source: Breast Cancer Res. 2013 Dec 19;15(6):R119. doi: 10.1186/bcr3588 (PMC4057522; doi:10.1186/bcr3588)
Supplement: Additional file 1 — Presents a description and illustration of the MMSDK method. [file bcr3588-S1.pdf]

Description of MMSDK Method

For MMSDK, short sequence tags tandem were obtained following digestion of genomic DNA with a methylation-sensitive mapping enzyme and a fragmenting enzyme. These tags were amplified, followed by direct, massive parallel sequencing and mapping to the reference human genome. The DNA methylation levels were inversely proportional to the number of tags mapped to the given genomic locus, since tags are only generated from DNA with unmethylated sites. The pipeline for MMSDK is illuminated in the figure below.

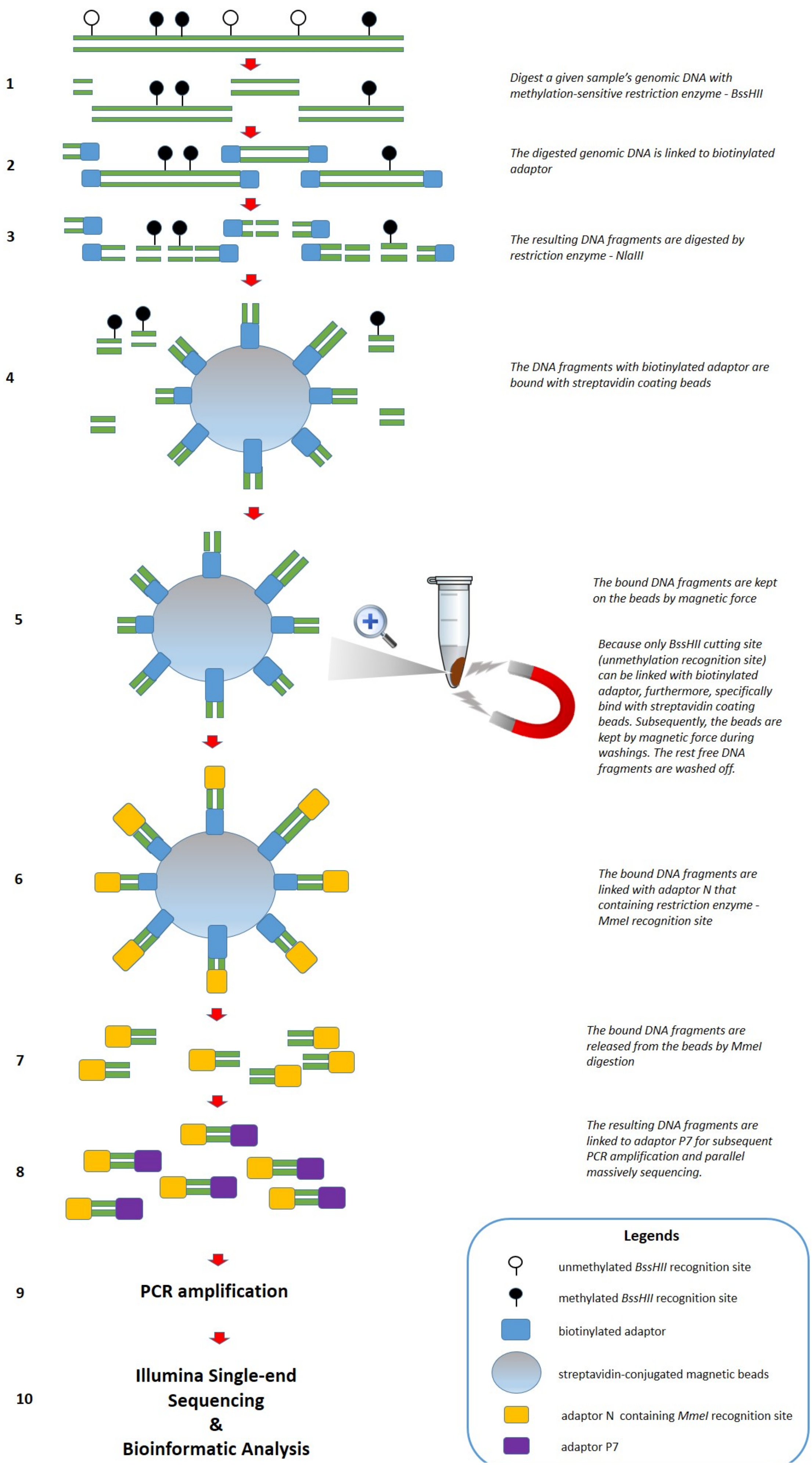
1
Digest a given sample's genomic DNA with methylation-sensitive restriction enzyme - BssHII
2
The digested genomic DNA is linked to biotinylated adaptor
3
The resulting DNA fragments are digested by restriction enzyme - NlaIII
4
The DNA fragments with biotinylated adaptor are bound with streptavidin coating beads
5
The bound DNA fragments are kept on the beads by magnetic force
Because only BssHII cutting site (unmethylation recognition site) can be linked with biotinylated adaptor, furthermore, specifically bind with streptavidin coating beads. Subsequently, the beads are kept by magnetic force during washings. The rest free DNA fragments are washed off.
6
The bound DNA fragments are linked with adaptor N that containing restriction enzyme - MmeI recognition site
7
The bound DNA fragments are released from the beads by MmeI digestion
8
The resulting DNA fragments are linked to adaptor P7 for subsequent PCR amplification and parallel massively sequencing.
9
PCR amplification
10
Illumina Single-end Sequencing
&
Bioinformatic Analysis
Legends
unmethylated BssHII recognition site
methylated BssHII recognition site
biotinylated adaptor
streptavidin-conjugated magnetic beads
adaptor N containing MmeI recognition site
adaptor P7
