# Supplementary material for: Integrative analyses of gene expression and DNA methylation profiles in breast cancer cell line models of tamoxifen-resistance indicate a potential role of cells with stem-like properties
Source: Breast Cancer Res. 2013 Dec 19;15(6):R119. doi: 10.1186/bcr3588 (PMC4057522; doi:10.1186/bcr3588)
Supplement: Additional file 2 — Lists the sequence of adaptors and primers used in this study. [file bcr3588-S2.pdf]

Sequence of adaptors and primers

Linker N: 5’-ACAGGTTCAGAGTTCTACAGTCCGACCATG-3’
3‘-CAAGTCTCAAGATGTCAGGCTG-5’

P7 Linker: 5’-TCGTAT GCCGTCTT CTGC TTG-3’
3’-NNAGCATACGGCAGAAGACGAAC-5’

PCR primers:
P5 long primer: 5’-AATGATACGGCGACCACCGACAGGTTCAGAGTTCTACAGTCCGA-3’
P7 primer: 5’-CAAGCAGAAGACGGCATACGA-3’

Sequencing primer
5'- CCGACAGGTTCAGAGTTCTACAGTCCGACCATG
