# Supplementary figures and images for: Integrative analyses of gene expression and DNA methylation profiles in breast cancer cell line models of tamoxifen-resistance indicate a potential role of cells with stem-like properties
Source: Breast Cancer Res. 2013 Dec 19;15(6):R119. doi: 10.1186/bcr3588 (PMC4057522; doi:10.1186/bcr3588)

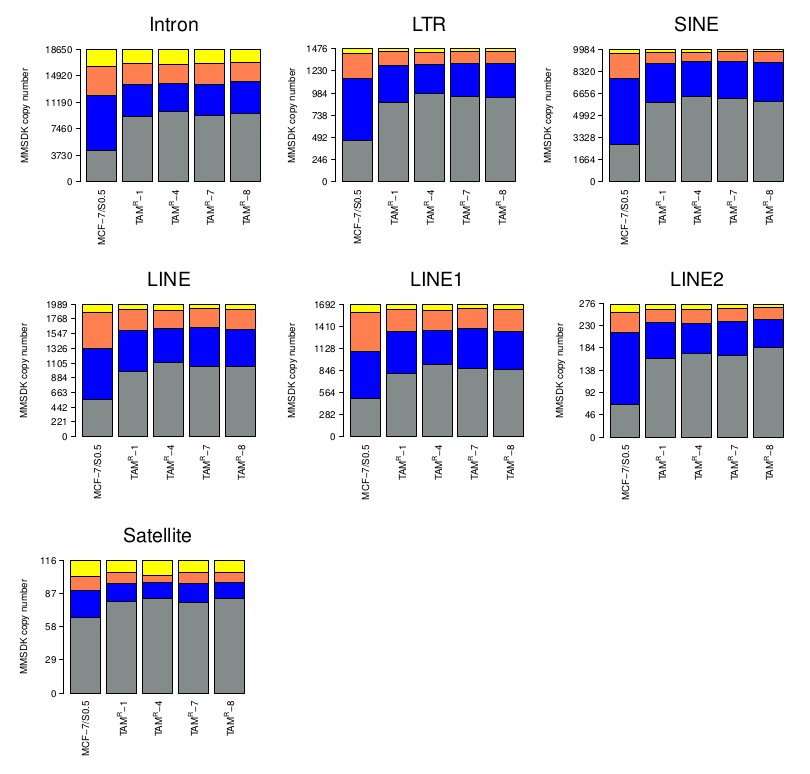

Supplement: Additional file 3 — Is a figure showing the distribution of DNA methylation levels of various genomic components in MCF-7/S0.5 versus TAMR cell lines. MCF-7/S0.5 shows low DNA methylation levels compared with TAMR cell lines in the different genomic components (intron, LTR (long terminal repeat), SINE (short interspersed elements), LINE (long interspersed elements), LINE1, LINE2, and satellite). The x axis shows the color-coded methylation states of CpGs for the MCF-7/S0.5, TAMR-1, TAMR-4, TAMR-7 and TAMR-8 cell lines. The mean methylation state of CpGs is categorized into very high (gray, 0 to 1 tag), high (blue, 2 to 10 tags), intermediate (orange, 11 to 100 tags), and low (yellow >100 tags). y axis shows the proportion of CpGs covered by methylation scores at low, intermediate, or high levels. Coordinates for genomic features were taken from the UCSC genome database and LINEs are defined by RepeatMasker. [file bcr3588-S3.jpeg]

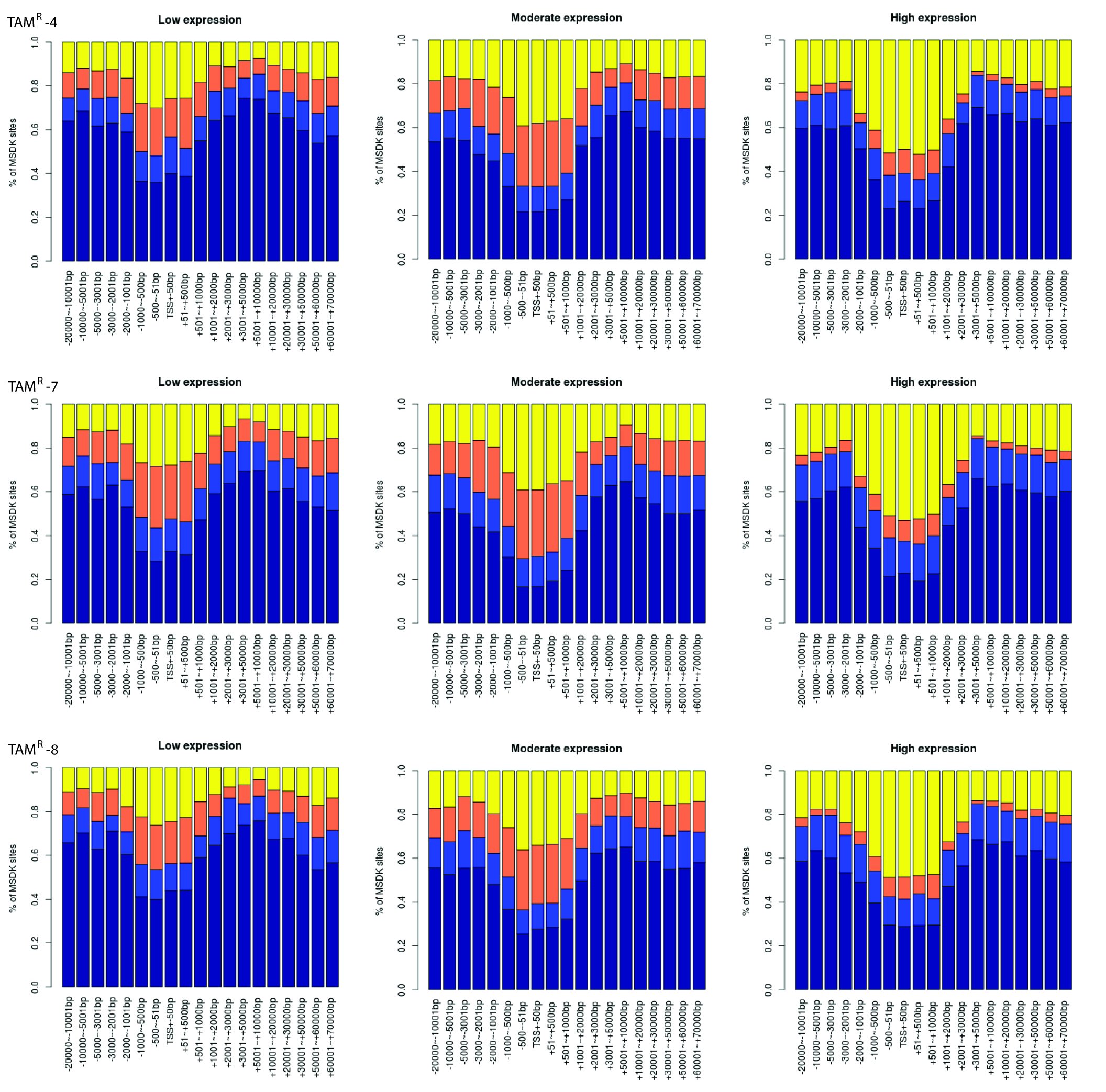

Supplement: Additional file 6 — Is a figure showing the relationship between DNA methylation and gene expression in TAMR-4, TAMR-7 and TAMR-8. An inverse relationship between DNA methylation and gene expression levels is noted. The expressed genes are grouped according to expression levels: low (left), moderate (middle) and high (right). Dark blue, light blue, orange and yellow represent extreme-high, high, medium and low DNA methylation levels, respectively. The x axis shows the genomic location relative to the TSS. The y axis shows the percentage of methylation for a given genomic location. [file bcr3588-S6.jpeg]
